# Overweight and obesity in young adult women: A matter of health or appearance? The Tromsø study: Fit futures

**DOI:** 10.3402/qhw.v10.29026

**Published:** 2015-09-22

**Authors:** Anne-Sofie Sand, Nina Emaus, Olaug Lian

**Affiliations:** 1 Department of Clinical Research, University Hospital of North Norway, Tromsø, Norway; 2 Department of Health and Care Sciences, Faculty of Health Sciences, UIT The Arctic University of Norway, Tromsø, Norway; 3 Department of Community Medicine, Faculty of Health Sciences, UIT The Arctic University of Norway, Tromsø, Norway

**Keywords:** Body size, weight balance, lifestyle, well-being, cultural norms, sensitivity, qualitative study

## Abstract

With the increasing number of overweight and obese people, there is a growing public health concern and focus on body size and lifestyle issues, especially in the media. Young adult women comprise a vulnerable group regarding issues of weight balance and appearance. The aim of the study was to examine the experiences of young women on how this focus influences their attitudes concerning weight changes, appearance, and health. We conducted 12 interviews with young women from two different weight groups about the attention on overweight issues. The results from the in-depth interviews were scrutinized through content analyses. The main findings indicate that young women experience a considerable focus on overweight issues with a trend towards appearance rather than health. Overweight and obesity are sensitive topics, and participants expressed strong views on the cultural definitions of normal body size and appearance. The squeeze between cultural norms and young women's perceptions of their own body and health was described as a possible negative factor influencing well-being as well as motivation for lifestyle changes. A more relaxed focus on overweight issues and especially on appearance is necessary when addressing weight-balance issues and lifestyle changes in young adult women.

During the past four decades, the increasing number of overweight and obese people has been described as epidemic and even pandemic (Swinburn et al., 2011). Overweight and obesity, especially in children, are now regarded as one of the main public health challenges by health authorities in most countries around the world and by the World Health Organization (WHO 2014a). The health consequences of overweight and obesity are disputed, but for some diseases, such as type 2 diabetes and sleep apnoea, overweight and obesity are regarded as dominant causes, and a number of other conditions and negative health markers are associated with increasing body weight (Swinburn & Bell, 2007). In addition, recent studies report how severe obesity is associated with greater all-cause mortality (Flegal, Kit, Orpana, & Graubard, 2013) and how bariatric surgery seems to lower this tendency (Arterburn et al., 2015). Overweight and obesity in childhood and adolescence are major concerns because it increases the risk of staying overweight or obese into adulthood with possible negative implications. Treatment and prevention in children and young people are consequently very important (Cunningham, Kramer, & Naryan, 2014; Gortmaker & Taveras, 2014).

Despite efforts made, so far no country has succeeded in reversing this trend (Roberto et al., 2015; Swinburn et al., 2011). The awareness and focus on overweight and lifestyle issues in public health campaigns, the weight loss industries, and, maybe, most importantly the media have been rapidly growing, especially since the early 2000s. Research has indicated that the public's perception of overweight and obesity is influenced by this focus (Johnson, Cooke, Croker, & Wardle, 2008). The young population might be even more sensitive to this influence (Friscoe, Houle, & Martin, 2010).

Changes in determinants of food preferences, physical activity, and body size perceptions in populations are still poorly understood, and more research is needed (Ogden et al., 2006; Swinburn et al., 2011). A review of papers examining weight gain in young women showed that in spite of evidence indicating that young women (18–36 years) gain weight at higher rates than women in any other age group, little is known about the determinants of weight gain in young adult women (Wane, Van Uffelen, & Brown, 2010). This lack of knowledge, suggested by others, formed the basis of our study and was, in combination with the serious public health concern, the reason for the following aim:

## Aim

The present study seeks to explore how young adult women from two weight groups, overweight and normal, experience the focus on body weight and lifestyle issues and how these experiences influence attitudes towards weight changes. In particular, we want to examine if the focus on body weight is an issue mainly related to health or to appearance, and to what extent overweight and obesity are perceived as sensitive issues.

## Overweight and obesity in the young population—what do we know?

The increase in the number of overweight and obese people was first noticed in the wealthy countries and initially affected the middle-aged population. However, younger age groups soon followed (Swinburn & Bell, 2007). Currently, in the United States, one-third of the country's children and adolescents are overweight or obese. However, several low- and middle-income countries around the world are now reaching similar numbers (Lobstein et al., 2015). In Europe, the corresponding number is more than 20% (Brug, Lien, Klepp, & Van Lenthe, 2010). In Norway, the most recent reports from the Norwegian Institute of Public Health point at obesity rates of more than 20% in the adult population with figures of overweight and obesity in adolescents between 20 and 30%. In children, one in six is overweight (Norwegian Institute of Public Health, 2014). It is furthermore worth noting that overweight and obesity in children and adolescents are more prevalent in the northern parts of Norway compared with the country average (Grøholt, Stigum, & Nordhagen, 2008; Norwegian Institute of Public Health, 2013). Although some recently published studies indicate that the obesity prevalence among children and adolescents might be leveling off in Australia, Europe, Russia, and the United States, the development is still a major worry among health managers, health workers, and politicians (Ogden, Carroll, Kit, & Flegal, 2014; Rokholm, Baker, & Sorensen, 2010; Stamatakis, Wardle, & Cole, 2010).

Current opinion suggests that prevention of and treatment for overweight and obesity should start early in life, during childhood, or even in pregnancy (Gillman & Ludwig, 2013). Adolescence and the transition into adulthood, however, are periods of crucial importance regarding the establishment of life-long lifestyle habits and skills. At this stage of life, the influence from peers, media, and surrounding factors might outweigh the influence from parents and family, especially when it comes to food habits (Lupton, 1996; Faw, 2014). Spending more time away from the family, and eventually leaving home, provide opportunities to experiment or even to rebel from childhood patterns of dietary preferences and eating habits. However, there is a lack of knowledge about lifestyle habits and not least the sociocultural determinants of these matters. Previous research indicates that girls and young women in the transition into adulthood are vulnerable to weight-balance issues (Wane et al., 2010). Some also experience depression because of weight problems, even when they are not overweight as defined by body mass index (BMI) classifications (Friscoe et al., 2010).

The growing awareness of overweight and lifestyle issues is apparent in society, in the media and in the fields of medicine, public health, and social research. For many adolescents and young adults, the perception of their own body image is of major importance for their psychological functioning and social relationships (Holsen, Jones, & Birkeland, 2012). With social media and the heavy use of pictures and picture sharing, it is hard to avoid being affected by the focus on body image. According to previous research, the relationship between body weight and perceptions of body shapes or images should be considered when planning prevention and treatment programmes (Bhuyian, Gustat, Srinavasan, & Berenson, 2003). Furthermore, attitudes on body size and weight are closely related to cultural norms (Wardle, Haase, & Steptoe, 2006).

“Body image satisfaction” has become a common term in research, frequently defined as the degree to which individuals are satisfied with their physical appearance, especially weight and shape (Holsen et al., 2012). Furthermore, body image dissatisfaction may be a possible predictive factor for depression, low self-esteem, and eating disorders (Friscoe, Houle, & Lippert, 2013; Holsen et al., 2012; Neumark-Sztainer, Paxton, Hannan, Haines, & Story, 2006). Few studies have investigated how a positive body image can be encouraged. One exception is the work of Frisèn and Holmqvist using an ongoing longitudinal Swedish body image study in adolescents. By interviewing the participants with a high degree of body satisfaction, several aspects emerged. Among them was the ability to evaluate the body more for its function than for its appearance, and to accept and learn to live with bodily imperfections (Frisèn & Holmqvist, 2010).

The concept of health, which is defined in many different ways, is complex and somewhat “slippery” (Blaxter, 2004, p. 148). The various definitions are always culturally and historically contingent as they are based on culturally defined norms about what we (in a given society) regard as normal and desirable. The WHO definition from 1946 intended to overcome biomedical views of health defined as absence of disease and introduced the definition of health as “a state of complete physical, mental and social well-being” (WHO, 2015). The definition is often regarded as an unrealistic ideal. Many scholars have suggested closer descriptions and definitions and Hans-Georg Gadamer's focus on the enigmatic nature of health is well known. Gadamer (1996) states that health is not only an individual experience, but a “condition of being involved, of being in the world, of being together with one's fellow human beings, of active and rewarding engagement in one's everyday tasks” (p. 113). In recent years, the meaning of health has been further expanded and described as culturally influenced and context bound. Health has also increasingly been linked to related notions of lifestyle, risk, and well-being (Nettleton, 2013). In this study, the age and sex aspects are highly relevant whenever health is mentioned or discussed.

Among others, the sociologist Nettleton (2013) claims that the focus on lifestyle in relation to health reflects broader social changes. Body maintenance and body management have become parts of our consumer culture. Attempts to avoid becoming fat and the tendency to maximize fitness may be more associated with physical attractiveness and accepted aesthetics than with physical health (Lupton, 2012). The emphasis on lifestyle and body maintenance as a way for young women to define and control themselves is not new, however. Striving for slenderness and self-management, women have been under cultural and biopolitical influence through long periods of history (Bordo, 1990; Jutel, 2001). In the present study, we had the chance to investigate experiences regarding body size, body perceptions, and lifestyle issues in young adult women within a secondary school setting in an urban area in northern Norway.

## Methods

To gain knowledge and deeper understanding of the phenomena we attempted to study, a qualitative research approach was chosen. This meant that we, in an interview setting, asked young women questions about experiences, perceptions, and opinions regarding overweight issues. To get access to people's experiences, thoughts, and values, we have to go beyond the answers that we can obtain from questionnaires and take into account the participants’ own experiences and their perceptions of body, health, knowledge, and existence (Dahlberg, Drew, & Nyström, 2008; Polit & Beck, 2008). By performing semi-structured interviews, we aimed at covering both the experiences of the single participant as well as opinions from the relevant sex and age groups on subjects connected to body weight and lifestyle matters. The questions were carefully planned and an interview guide was developed, ensuring that all the relevant aspects were covered. Through semi-structured interviews, we explored experiences and descriptions of the participants’ lived everyday world regarding issues connected to the scope of the study (Kvale & Brinkmann, 2009). The data from the interviews were consecutively analysed by using content analysis.

### Participants and recruitment

Participants were recruited from a school-based population survey, Fit Futures—which is part of The Tromsø Study (2015). Students from two municipalities were invited to participate in the survey twice, during the first year (at the age of 15–16 years) and the last year (at the age of 18–19 years) of secondary school. The attendance rates of 93 and 77%, respectively, were good, with around 1000 attendees in each of the surveys. The majority of the students lived in the urban area of Tromsø town with its approximately 72,000 inhabitants. The surveys consisted of anthropometric measures, physical examinations, blood samples, and a comprehensive online questionnaire on background data and lifestyle issues. Results from the survey, including the participant's BMI, were sent as a letter to all the participants after attendance.

For the present study, two groups of women from the Fit Futures cohort were invited. The substantial sex differences regarding overweight and lifestyle in adolescents and young adults are well known (Holsen et al., 2012; McCabe, Ricciardelli, & Ridge, 2006; Nilsen, Krokstad, Holmen, & Westin, 2010; Wardle et al., 2006). The differences are apparent in several ways, for example, in food choices and eating habits (Nilsen et al., 2010). Furthermore, messages from sociocultural agents, such as family, friends, and the media, differ in quantity and substance between the sexes, and girls are regarded as most vulnerable (McCabe et al., 2006). Adolescent girls also report persistently lower body satisfaction than boys (Holsen et al., 2012). Because of the well-documented sex differences regarding the most relevant aspects of the present study, we narrowed our focus to young women.

The age range in our sample was between 18 and 20 years at the time of the interview. The sample consisted of two different groups categorized according to BMI levels as measured in Fit Futures 2. Normal weight is defined as BMI between 18.5 and 24.9 kg/m^2^; overweight as BMI between 25.0 to 29.9 kg/m^2^; and BMI≥30 kg/m^2^ is regarded as obesity, all according to the WHO (2014b).

One group of participants was moderately overweight or slightly obese, with a BMI from 27.0 to 32.9 kg/m^2^. In the other group, participants within a normal weight range with a BMI from 18.5 to 24.9 kg/m^2^ were selected. We invited two groups with different BMIs in order to explore experiences and perceptions from a more complete range of perspectives regarding the participant's own body size. However, we excluded those who were underweight or severely obese.

Eligible participants received information about the study (informed consent in two copies) with an invitation to participate sent by staff from UiT the Arctic University of Norway and The Tromsø Study/Fit Futures. The participants received a gift voucher (value 200 NOK, equivalent to 25 EUR) as compensation for travel expenses and time spent. The response rate for the present study was disappointingly low. One possible explanation for this was that the participants could be less motivated for participating in more research just after attending the survey. Most of them also had or were about to finish school and some had moved on to study or work in or out of town, which could influence their ability to attend. Another possible explanation could be that weight issues are a sensitive topic. Eventually, 6 participants from each weight group, in total 12 participants, were included in the study that we performed between June 2013 and February 2014.

### Interviews

Semi-structured, individual in-depth interviews based on an interview guide were performed. The interviews were audiotaped and lasted between 43 and 81 min, with a mean duration of 56 min. The first author conducted the interviews at the Clinical Research Unit at the University Hospital of North Norway. The interviews were preceded and concluded with some informal small talk to help the participants relax and to try to normalize the conversation. The initial questions were rather open with small adjustments like this: “What is your opinion about the attention and focus on overweight and lifestyle issues around you, in the media, at school, at home and in the society in general?” After this introduction, the participants were asked questions from the interview guide covering the following main topics: nutrition and eating habits, physical activity in school and leisure time, sleeping habits, stress in everyday life, lifestyle as a concept, media influences, and the 24/7 lifestyle. We asked about their actual habits as well as their opinion regarding these matters. The interview guide was used as a tool to ensure coverage of all the topics but not always in the same succession. Other relevant issues that turned up during the interview were explored. The interviews were concluded by asking if anything relevant had been left out of the conversation, to make sure that the participants’ point of view was included in the data.

### Analysis

The first author performed verbatim transcriptions and analysed the interview texts using qualitative content analysis. The research questions implied rather multifaceted and sensitive phenomena. Messages, symbols, and information between people and between people and mass media are important parts of the picture, making content analysis a suitable tool for analysing the data (Vaismoradi, Turunen, & Bondas, 2013).

For each of the interviews and throughout the analysis, meaning units were defined as fragments of text containing information about the research questions (Graneheim & Lundman, 2004). The units were organized by colours after identification of preliminary themes connected to the research questions. Furthermore, the units’ condensed meanings were studied, both as descriptions close to the text and as condensed meanings and interpretations of underlying meaning. These condensed meaning units were then categorized into recurring subthemes and main themes with regards to the study objectives, as shown in Table I.

**Table I:** Appearance versus health.

Meaning unit	Condensed meaning unit, description close to text	Condensed meaning unit, interpretation of underlying meaning	Sub-theme	Theme
I think that there's not enough focus on the health aspects of being overweight … there's a big difference between focusing on your health and focusing how you look …	Being disappointed by the focus	Frustrated by the angle of the focus	Biased focus, missing the health aspect	Appearance versus health, biased focus
It's more important to me to eat healthy than to actually become thin …	Being healthy is more important than being thin	Opposing pressure on appearance	Biased focus, too much on appearance	
The media is focusing on the consequences rather than the reasons why people develop overweight …	The focus should be more on why people develop overweight	Disappointed by the media	Biased focus	

### Example of analysis process

In order to achieve trustworthiness, the various steps of analysis were scrutinized and discussed by all the authors in accordance with concepts of credibility, dependability, and transferability. We paid special attention to individual and group similarities and differences regarding experiences and expressions related to emerging trends and patterns. By presenting the variety of experiences from the participants and simultaneously focusing on the research question, we tried to enhance the credibility of the study. The data gathering was accomplished through a limited amount of time (8 months). The data were consecutively transcribed and analysed to increase dependability.

The study was a population-based school survey. The participants represented the same sex, age group, and geographical area, and they were or had recently been in upper secondary school. Contextual background and demographic setting can be important when it comes to the degree of transferability. We will return to the matter of transferability in the “strength and shortcomings” section. Through the analysis, we tried to focus on novel and unanticipated themes and issues that challenged our pre-understanding.

### Ethical considerations

The study was approved by the Regional Committee for Medical and Health Research Ethics; REC North (2012/1621). Eligible participants received written and oral information about the aim of the study, the length of the interview, and procedures for confidentiality before accepting to participate. The information also stated that participants should not expect any advantages or inconveniences, as long as discussing overweight matters did not make them feel uncomfortable. Participation was presented as voluntary, and declining to participate would not have any consequences for the individual.

Those who agreed to participate returned one signed consent form in a prepaid envelope or contacted the first author by e-mail. Those who had not signed the informed consent in advance did so when attending the interview.

## Results

The findings are presented through three essential main themes, reflecting emerging aspects related to the aim of the study. The participant's quotes are followed by pseudonyms, but with the actual weight groups.

### The magnitude of the focus—health, appearance, and fitness issues

The participants described their experience with the focus on overweight and lifestyle issues in various situations and contexts in both similar and different ways. There were variations both within and between the two groups. Participants from the overweight group described it like this:

I think it is way too much … and it is all about being thin and perfect … I think that everybody is perfect in their own way. (Mia, overweight group)

It's a fact that overweight persons in general have lower self esteem … and if the focus had not been that strong, it would not be such a big problem. (Lisa, overweight group)

I think it is huge (the focus) … I have been called fat and overweight … and I'm not the biggest person … I'm reminded of my state every day. (Mona, overweight group)

These young women clearly felt the attention on overweight issues as challenging. Participants from the normal weight group tended to describe the focus as part of the general lifestyle and healthy living issue:

There is in general a focus on lifestyle issues such as dieting … but maybe not enough on overweight as a problem among young people. (Liv, normal weight group)

Yes, I think there is a strong focus on being healthy … it's like you have to be lean to be successful. (Sarah, normal weight group)

The magnitude of the focus was obvious, judging from the descriptions from most of the participants. There were, however, differences and variations in the way it was described. The participants in the overweight group seemed to have reflected more on the issue, and appeared to be more sensitive and vulnerable to the impact of the focus. The most prominent finding was that for most of the participants from both weight groups, the focus on overweight and lifestyle was considered to be mainly an issue of appearance. This was most obvious when the participants described the nature of the focus, as seen in these quotations from participants in the overweight group:

I think that there's not enough focus on the health aspect of being overweight … there's a big difference … focusing on your health and focusing on how you look … there's not so much talk about consequences …. (Lisa, overweight group)

And, continuing her thoughts about health:

I might not be the healthiest person, but I do not have unhealthy perceptions of body and health.

Furthermore:

It's more important for me to eat healthy and to be physically active than to become thin. (Sophie, overweight group)

These quotes illustrate spontaneous reactions when asked about the nature and the impact of the focus. These participants are protesting against the ways overweight is presented as a problem. Furthermore, the participants are questioning the reasoning behind why weight reduction is so important. Is it really a matter of health or is it a matter of appearance? Participants from the normal weight group also expressed strong views on this matter:

It is important to fight overweight … but it is more important to live healthy. (Hannah, normal weight group)

Women from both weight groups thought that more focus on health issues related to overweight and obesity was needed. The media's attention to overweight and lifestyle issues was based on appearance and body image rather than the health aspects, and was regarded as disappointing and even provoking.

Several participants mentioned the focus on fitness and building muscles. This was most distinct in the normal weight group:

Right now I think that the focus is shifting towards an attention on exercise and fitness … you're supposed to be active … build muscles … before it was important to be thin … now it's important to be strong! (Hannah, normal weight group)

The focus on overweight was bigger before I think … Nowadays, there's more of a focus on fitness and nutrition. The public is divided between those who exercise and those who don't. (Elisa, normal weight group)

The importance of looking fit rather than just lean emerged as a new and dominant subject. Five of the participants from the normal weight group felt the focus was more of a fitness issue than an overweight issue, describing it as a trend. Most of these women were physically active at a high level, and they emphasized that this was their personal opinion. The statements about a public divide over the issue of exercise habits are worth noting. Belonging to “an exercise community” seemed to be an important motivation for physical activity for some young adults, as an identity issue. In the overweight group, only one of the participants highlighted the fitness issue:

Right now it's like you have to be both lean and strong, but it changes all the time (about media influence). (Mona, overweight group)

This expression reflects both distance and ambiguity concerning the matter of fitness and suggests that this phenomenon is just another wave or temporary fashion trend.

### The power of definition and being proud of yourself

Definitions of normality regarding body weight and sizes emerged early in the course of several interviews, often as questions about or comments on what is considered normal weight and who decides. Participants from the overweight group commented like this:

I do not agree with the definition of overweight set out by the media … you almost have to be anorectic to be accepted … but I don't care much about this. (Sophie, overweight group)

I get so tired of it! … There's a lot of fuss about it really … I will rather read about people who dare not to be ‘perfect’ … like ‘you go girl’! (Mona, overweight group)

There has been attention (from a family member) on my overweight since kindergarten actually … and it has really damaged my self-confidence. (Helen, overweight group)

These blunt statements referred mainly to signals from the media, friends, and family, which seemed to be the most important agents of influence. Several participants from the overweight group described receiving results from the survey and the invitation for the present study as signals from others about their health in general and weight issues in particular:

I felt like I was labeled … most people who are overweight are aware of it … but other people telling you the facts feels even worse. (Lisa, overweight group)

I did realize it … the overweight … and I had already decided to do something about it, so getting the message was a little hard, but still fair. (Helen, overweight group)

The first of these excerpts shows how a participant from the overweight group was deeply offended by the message. The quote that follows shows a reaction that is far more relaxed and accepting. The reactions varied in strength, and some were struck and even hurt by the feedback from the survey.

As the interviews unfolded, several of the women from this group elaborated on how the general focus on overweight affected them:

I don't know … I think maybe we should have paid even more attention to it … I mean, people just sit there … knowing about their overweight, but not knowing what to do. (Kate, overweight group)

They describe overweight as a ‘bad thing,’ but I mean … the risk of disease is increasing … it's not healthy to eat too much … so what they say is actually the truth. (Helen, overweight group)

These participants pointed out that they were aware of the fact that they were overweight and that this could possibly have a negative impact on their health. They maintained that the focus on overweight and lifestyle issues was not entirely without purpose and good intentions. The participants from the normal weight group were less judgmental about the focus, but some stated that more attention to preventing overweight was important. Women from both groups noted that female models with a more “realistic” appearance in terms of “not too skinny” were absent from the media, and they considered this an important signal. Signals from the surroundings about overweight as something you should fight seemed to make participants from the overweight group feel ambiguous about their appearance. One of them had an open-minded reflection on her body and appearance:

I really like my big thighs … to show that I am a woman! (Saying this with a good laugh following). (Mona, overweight group)

In this way, she described her body in a positive way and with pride, as she perceived her body shape as a symbol of her own femininity. She continued:

There is a lot of talk about overweight … it can be dangerous … you can even die … so of course it is important to talk about it …

About her own experiences she stated:

… but sometimes it becomes too much really … you're beginning to feel like a bad person … like nothing … a big lump of fat.

In this way, Mona described the bad feelings and even the shame she sometimes felt. Furthermore, she felt rather ambiguous about trying to lose weight. When discussing the health aspect she expressed a lot of ambiguity, but concluded her reflections rather bluntly and said she was unwilling to make the effort needed to lose weight:

I know I should do something to lose weight, but I really just don't bother … I am glad that we are different in shapes and sizes … would be boring if we were all the same. (Mona, overweight group)

Lisa from the same group phrased it like this:

The media focus has changed somewhat, there is more attention on accepting yourself as you are … or maybe it is just me getting older? … (laughs)

Continuing:

… a lot of people stand up against the focus on body image and appearance … and I think that this will alter the focus … but it will take some time.

These two participants expressed opposition against the focus on body weight and body image. There seemed to be a widespread belief among the participants in both groups that there would be future changes regarding the public perception of body images and the ideal body.

### Overweight and obesity as a sensitive theme

Several participants in both weight groups declared problems with discussing overweight and obesity, but this was most obvious in the normal weight group:

Overweight problems are not a big issue among youths … it is a sensitive theme … and I do think that opening up the subject would make it easier to be overweight and to get motivated for weight loss…. (Liv, normal weight group)

Liv added that this was something she had experienced herself during a time of weight gain, and she continued:

I'm so lucky to be in a group of friends who can talk about everything … including our shortcomings. (Liv, normal weight group)

The more openness the better (regarding both under- and overweight), openness makes it easier to bring on change … it makes it easier for other people to help. (Hannah, normal weight group)

I think it's maybe a little more accepted to be overweight these days … but still … it IS a sensitive subject … you don't know what to say for fear of hurting people. (Ellen, normal weight group)

Furthermore, another participant from the normal weight group described it as a theme one would actually try to avoid:

People are afraid of being labeled overweight (regarding talking about overweight issues) … even if they don't have that problem at all. (Emma, normal weight group)

The latter expression can be interpreted as another sign of a perceived divide in the population regarding weight and lifestyle issues in young women. Sensitivity to other people's perceptions seemed to be of considerable importance when discussing weight and lifestyle issues.

There were similar signs in the overweight group, like in this expression:

I haven't told my friends about my participation in this study … they really don't need to know … I think that this could change the way they look at me and make them think of me as an overweight person. (Lisa, overweight group)

These thoughts show her ambiguity about participating in the study. The fear of being labelled overweight actually made her think twice. Another description of the sensitivity issue appeared when we asked about attending physical exercise lessons in school:

Girls tend to judge people from what they see … and this makes you anxious about talk and gossip. (Kate, overweight group)

Furthermore, participants from the overweight group pointed to the need for more openness:

Body ideals are not discussed enough, the ideal body is still very lean. (Helen, overweight group)

I really like to discuss overweight matters … you hear people saying really odd things. (Mona, overweight group)

In the overweight group, experiences and attitudes regarding talking about or discussing overweight issues were quite varied. The difference between the two weight groups on this point was, however, notable. The calls for “opening up the subject” by participants from both groups are important signals and underscores that the sensitivity of the subject can be an inhibitor for both well-being and motivation for lifestyle changes.

### Summary of the results

In this study, whenever overweight issues were expressed through media and in everyday life, appearance was emphasized over health. This observation was followed by expressions of disappointment and worry. Previous research indicates that girls and young women face a substantial number of messages about their bodies, from friends, family, and media (Frisén & Holmqvist, 2010; McCabe et al., 2006), and this was confirmed in our study. Most of the participants disapproved of the exaggerated focus in the media, which they perceived as biased.

The results also highlight the power of definition of normality and the importance of being proud of yourself, independent of your body size. Most, but not all, of the participants felt that the issue was sensitive.

## Discussion

We will now turn to the discussion of the results, with a closer look on the notions of cultural norms, health, defining normality, and finally sensitivity.

### Cultural norms

The cultural norms regarding appearance, as exemplified by the idealization of a strong and fit body, or in general a lean body, were by some participants from the overweight group described as damaging for their self-confidence and well-being. The focus on appearance and body image was followed by frustration over the narrow definition of normality. These cultural norms and expectations, often transmitted through the media, but also through friends and family, were clearly present in the findings. Participants from both weight groups stated that more diversity regarding weight and body shapes in female role models would be highly valued. Some quotes revealed confusion and frustration regarding the pressure on women to adjust to feminine ideals communicated by the media. Such ideals have been shifting through the years, from the “hour-glass” shape to the feminine body as disciplined and slender (Bordo, 1990; Rich & Evans, 2009).

We found examples of the impact of cultural norms and how they can cause a feeling of confusion and pressure. One of the participants from the overweight group said she was proud of her curvy lines, which she perceived as feminine. She was, however, receiving contradictory signals from her surroundings, especially the media. For her, this caused frustration and mixed feelings towards weight loss and lifestyle changes in order to lose weight. Another example is how fitness emerged as a new and important issue. Some of the participants, mainly in the normal weight group, stated that this was how “everyone should look” these days. One of the participants even referred to a “public divide” regarding exercise and fitness matters. The results suggest that this was not only because practicing physical exercise supposedly made them look good but also as proof of healthy living and fashionable lifestyle choices. In this way, physical activity as lifestyle, and consumer issues, emerges as cultural codes and ways of differentiating social groups as described by, for example, Bourdieu (1984) and Nettleton (2013). The way fitness is mentioned by some of the participants reveals a specific view of lifestyle choices and the physical presentation of the body. The right lifestyle choices are perceived as important, not only as investments in health and aesthetic aspects but also as a matter of social identity and distinction (demarcation) against other groups.

### The notion of health

Participants from both weight groups felt that health in a wider perspective was more important than being within the range of normal weight. When elaborating on the notion of health, this was often described in terms of overall well-being and confidence, as well as good eating habits and being physical active. A good perception of your own body, as well as a good self-esteem, was expressed by the participants as important factors of healthiness. Interestingly, well-being defined this way is connected both to the matter of body image satisfaction and to the more traditional perceptions of health as described by Nettleton (2013). It is known from previous research that young people's perspectives on health might differ from those of the adult population, health workers, and health authorities. Spencer (2014) highlights the importance of recognizing this fact when exploring empowerment and health concerns among young people. The importance of defining health in a more positive way, even as “having fun,” as found in Spencer's work, is in line with our findings. In this way, young people's perception of health seems to go far beyond just absence of disease. The concept of health seems to be highly context- and age-sensitive (Kristensen & Køster, 2014; Spencer, 2014).

There was, however, a tendency for some of the overweight women to justify the focus on overweight and lifestyle issues for more traditional health-promoting reasons, such as prevention of disease. Furthermore, this could be interpreted as uncertainty about their own overweight and weight loss attempts. These participants stated that they felt that they should lose weight for health reasons but were unable or unwilling to bring on the necessary changes. Concerns about health, combined with the obvious, sometimes frustrating, and almost exhausting focus on appearance, are connected to considerable ambivalence in young women, and this could possibly prove a negative influence on motivation for lifestyle changes (Chung, Sherman, Goodman, Bickham, & Rich, 2013; Neumark-Sztainer et al., 2006).

### Questioning the definition of normality

As shown in the results section, both disappointment and frustration surfaced when perceptions of normality regarding body sizes were discussed. Strong voices for a more accepting view of the diversity of body sizes and shapes emerged in the data, and the importance of being proud of oneself regardless of appearance was frequently mentioned.

Results from several other studies indicate that girls tend to be more sensitive to messages about body image and have lower degrees of body satisfaction than boys through adolescence (Frisén & Holmqvist, 2010; Holsen et al., 2012; McCabe et al., 2006; Neumark-Sztainer et al., 2006). Body maintenance as a means of obtaining good health as well as sexual attractiveness has been a theme among researchers in the social sciences in recent decades (Bordo, 1990; Nettleton, 2013). The findings in our study confirm that young women are highly sensitive to the fluctuations in fashion, body ideal, and lifestyle.

The interview phrases about being proud of one's own body also confirm suggestions from previous research that given the opportunity to discuss body image, girls or young women tend to mediate their negative feelings (McCabe et al., 2006). The importance of body satisfaction for overall well-being, especially in young women, is also well known (Friscoe et al., 2013; Frisén & Holmqvist, 2010; Neumark-Sztainer et al., 2006).

### Sensitivity

Overweight was not presented as a hot topic in everyday conversations in our study, but rather as a somewhat surprisingly sensitive issue. All the women in the normal weight group described it as difficult to discuss overweight with people who are overweight. Fear of practicing prejudice or even hurting people were mentioned as reasons for this. However, they maintained that, in their opinion, sensitivity and lack of openness could make it even more difficult to be overweight. On the contrary, only two of the women in the overweight group clearly expressed difficulties with discussing the matter. The other women in this group were more relaxed, and two of them were even enthusiastic about discussing overweight matters. However, they describe this as a gradual development that matured over time through adolescence. There have been signs of changing perceptions and a normalization of overweight in our society with prevalence of overweight and obesity reaching 50% in the adult population in countries such as the United Kingdom and the United States (Johnson et al., 2008; Wardle et al., 2006). For the Fit Futures Survey, the prevalence of overweight and obesity was about 23% (Winther et al., 2014), and changing perceptions of overweight as a consequence of social comparison cannot be ruled out in the present study. Still, the fact that overweight for most of the participants was a sensitive theme in conversations contrasts the description of an omnipresent media focus on the subject.

## Conclusion: is overweight and obesity a matter of health or appearance?

In our study, the main findings were a substantial focus on overweight and obesity coupled with an attention bias towards appearance rather than health in the perceptions of young adult women. Overweight and obesity were also regarded, to a large extent, as sensitive issues in everyday life. As a result, negative effects on self-confidence and general well-being were described by the participants in the overweight group. Furthermore, signs of ambivalence and confusion about how to handle weight issues were found in both weight groups. Wider definitions of health in terms of overall well-being and self-confidence were also found.

The emphasis on well-being and the possible link to both appearance and health are interesting aspects that should be further explored in future research.

In our opinion, the study underscores the need to reduce the focus on appearance and body image when overweight issues are communicated or discussed with young adult women. The findings indicate that this could have positive effects on their well-being, especially for the overweight and obese. For some of the participants, the pressure on appearance and body image led to a feeling of being squeezed between cultural norms and their own perceptions of body and health. Furthermore, this was described as discouraging regarding motivation for a healthier lifestyle. Thus, a more relaxed attitude towards appearance, especially body image, and more focus on health and general well-being when health authorities, health workers, or other relevant professionals address weight-balance issues in young adult women is needed.

### Strengths and shortcomings

A school-based population study formed the basis of the sample, ensuring that age, living area, and school progression were similar for all participants. Interviewing young women from both an overweight and a normal weight group from the same cohort revealed both differing and similar views, and broadened the picture on how the focus on overweight and lifestyle affect young adult women.

The small sample could be a limitation, and the low response rate could imply inclusion of a selected sample. This could influence the results, especially regarding the sensitivity issue.

There is always a question of transferability regarding geographical location, and known and unknown contingencies, such as sociocultural aspects. In the Western world, however, mass media and modern communication means that many references and cultural values to a large extent should be comparable.
